# Genetic Interactions Show the Importance of rRNA Modification Machinery for the Role of Rps15p during Ribosome Biogenesis in *S. cerevisiæ*

**DOI:** 10.1371/journal.pone.0010472

**Published:** 2010-05-03

**Authors:** Clément Bellemer, Pauline Chabosseau, Franck Gallardo, Pierre-Emmanuel Gleizes, Guillaume Stahl

**Affiliations:** 1 Laboratoire de Biologie Moléculaire Eucaryote, Université de Toulouse, Toulouse, France; 2 Laboratoire de Biologie Moléculaire Eucaryote, CNRS, Toulouse, France; Institute of Protein Research, Russian Academy of Sciences, Russian Federation

## Abstract

Rps15p, an essential ribosomal protein, was previously shown to be critical for nuclear export of small subunit pre-particles. We have designed a synthetic lethal screen in *Saccharomyces cerevisiæ* to identify its genetic partners and further elucidate its role during ribosomal biogenesis. Our screen revealed interactions with mutants affected at various stages during ribosome biogenesis, from early nucleolar steps to nuclear export. Mutations were identified in genes encoding proteins involved in early ribosome biogenesis steps, like the small subunit processome component Utp15p, the 90S pre-ribosome factor Slx9p and the H/ACA snoRNP core protein Nhp2p. In addition, we found a synthetic lethality with *BUD23*, a gene encoding a methyltransferase involved both in rRNA modification and small subunit nuclear export. Interestingly, deletion of snR36 or snR85, two H/ACA snoRNAs that direct modifications close to Rps15p's binding site on the rRNA, produces mild and opposite effects on growth in an rps15 hypomorphic background. These data uncover an unreported link between a ribosomal protein and rRNA modification machinery.

## Introduction

**Figure 1:**
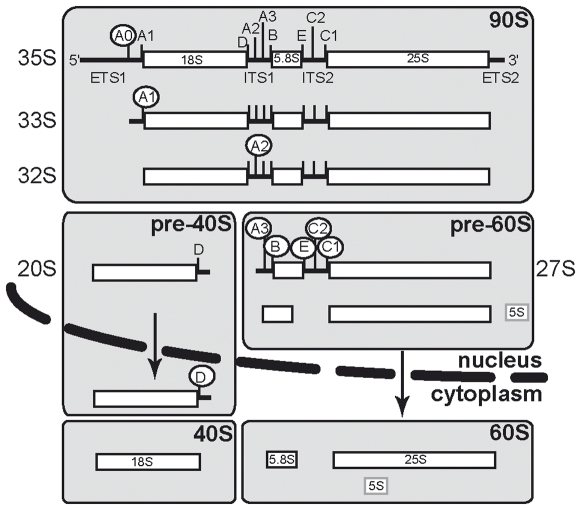
Schematic overview of yeast ribosomal biogenesis pathway. White circles indicate the next processing steps. Gray boxes indicate different processing intermediates. Dashed line represents the nuclear envelop, and arrows the export process. rRNA species names are indicated on the sides. 5S rRNA is transcribed independently and then joins pre-60S particles.

Ribosome biogenesis in eukaryotes is a complex process that takes place, for most of it, in the nucleolus, a specialized domain of the nucleus. RNA polymerase I synthesizes a large ribosomal RNA precursor (pre-rRNA) that includes three out of the four ribosomal RNAs constituting the ribosomal subunit, *i.e.* the 18S, 5.8S and 25S rRNAs in yeast. Conversion of this large precursor to the mature species involves sequential removal of flanking and internal sequences, the external (ETS) and internal transcribed spacers (ITS), through action of endonucleases and exonucleases (Fig. 1; for a review, see [1]). In addition to cleavage, the pre-rRNA is subjected to a series of nucleotide modifications, mostly ribose 3′-O-methylations and pseudo-urydilations. These modifications are catalyzed by small nucleolar ribonucleoparticles (snoRNPs) through specific base-paring between their RNA component (snoRNA) with the surrounding of the position to be modified. These RNA processing steps are intimately coupled to the assembly of diverse proteins with the precursor RNAs, which starts as soon as transcription is initiated. These proteins include the 79 ribosomal proteins, which remain associated to the mature subunits, as well as a large number of trans-acting factors. The large ribonucleoproteic particle thus assembled in the early part of the pathway, or 90S pre-ribosome, is composed mainly of so-called UTP proteins (U-Three Particle), which associate to the U3 snoRNP [2]. After participating in the early 18S rRNA maturation steps, these factors are released from pre-ribosomal particles when paths to form the precursors to the 40S and the 60S ribosomal subunits separate after A2 cleavage (Fig. 1).

The multiple steps of this process are highly organized in the cell nucleus as indicated by the dynamics of the nucleolus, the formation and morphology of which strictly depend on the activity of ribosome biogenesis [3]. The late steps of ribosome biogenesis, however, sequentially take place in the nucleoplasm and in the cytoplasm. For instance, the 40S ribosomal subunit, when exported from the nucleus, contains the 20S pre-ribosomal RNA which 3′-end maturation in the cytoplasm yields the mature 18S rRNA. The determinants of the nuclear export of the 40S subunit are still poorly known. The exportin Crm1p is necessary [4], but although potential interactors of this exportin in the pre-40S particles were described, no critical binding site or essential adapter with the pre-40S particles has been found yet [5]. By screening yeast strains expressing sub-optimal levels of specific ribosomal proteins, we identified the ribosomal protein Rps15p as a particular actor of nuclear export of the pre-40S particles [6], a function conserved in mammalian cells [7]. Depletion of Rps15p provokes retention of the pre-40S particles in the nucleus without affecting the upstream RNA processing steps. It is tempting to speculate that Rps15p interacts with proteins involved in nuclear export of the pre-40S particles, like nuclear export factors or nucleoporins. Alternatively, shielding of a particular domain in the ribosomal RNA could be critical for nuclear domain, as already proposed for other pre-40S particle components like the transacting factor Rrp12p [8] or other ribosomal proteins whose depletion slows down nuclear export [9], albeit not as strongly as in the case of Rps15p.

Here, either through a random screen or starting from an educated guess, we have looked for mutations causing synthetic lethality with a thermosensitive allele of *RPS15* to find genetic interactors potentially involved in nuclear export. Our study reveals an unexpected link between *RPS15* and rRNA modification machinery.

## Results

### Screening of *RPS15* genetic partners

**Figure 2:**
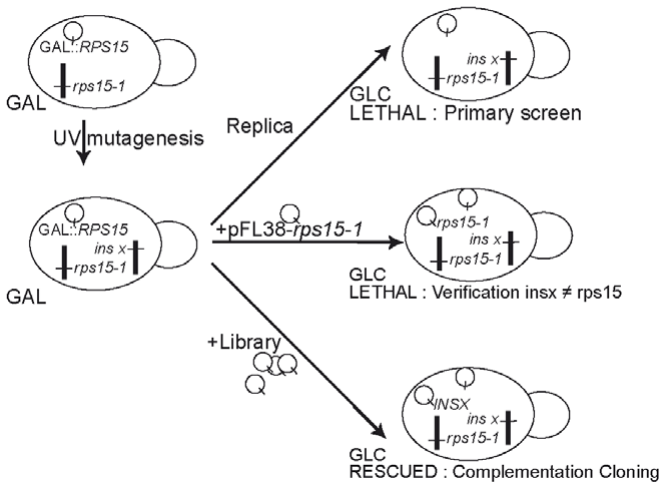
Schematic view of the synthetic lethal screen used. GAL, galactose containing media; GLC, glucose containing media, used to repress the GAL promoter. Bold lines represent yeast chromosomes; circles represent yeast episomes.

To ascribe precise functions to Rps15p and identify its partners, we designed a synthetic lethal genetic screen based on a yeast hypomorphic mutant *rps15-1*, thermosensitive at 37°C. At 25°C, this mutant has a generation time doubled relative to the WT strain, indicating a defect even at permissive temperature. We identified mutants worsening the phenotype associated with the *rps15-1* allele, by isolating synthetic lethal mutants at 25°C. Permissive conditions are provided by a conditional expression of wild-type *RPS15* from a galactose-inducible/glucose repressible promoter, while the mutant *rps15-1* allele is located at the chromosomal locus, expressed via its own constitutive promoter (Fig. 2). After UV mutagenesis, we isolated 8 strains bearing a mutation synthetic lethal with *rps15-1*, that grew on galactose but not on glucose. Loss of function at the chromosomal *rps15-1* locus was excluded since synthetic lethality was not rescued with plasmid pFL38-*rps15-1* (Fig. 2). The strains satisfying this secondary screening were called Ins3, 5, 7, 9, 11, 13, 15 and 17 (Ins is Not S15).

### Complementation Cloning of the *ins* mutants

With the exception of Ins3, which happened to be sterile, all Ins mutants were backcrossed with an *rps15-1* strain and proved to be recessive. By transforming a yeast genomic library (kindly provided by P. Thuriaux) and selecting on glucose at 25°C, reproducibly complementing clones were obtained for Ins9 and Ins15. Plasmids complementing Ins9′s growth on glucose, and remaining thermosensitive, all contained genomic regions encompassing the *NHP2* gene (*YDL208W*). Nhp2p is essential for function of H/ACA-type snoRNPs [10], which serve as guides to pseudo-uridylate about 46 U residues on rRNAs, including thirteen in the 18S rRNA. Plasmids complementing synthetic lethality in strain Ins15 contained *UTP15* (*YMR093W*), an essential gene coding for a component of the U-Three Particle, as defined by Baserga and co-workers [2]. Sequencing of *nhp2* in the Ins9 strain identified a TG deletion near the end of *nhp2* ORF, leading to a frameshift mutation 20 codons before the end of the coding sequence. This is predicted to change the C-terminal sequence, and further lengthen the protein by 11 amino-acids. In the Ins15 strain, we found that *utp15* translation start codon was mutated from AUG to AUU. As *UTP15* is an essential gene, translation initiation is likely to proceed in some other unidentified way, which might still support poor growth in the presence of WT Rps15p, but become lethal in an *rps15-1* background. The two mutated alleles *ins9* and *ins15* were thus renamed *nhp2^ins9^* and *utp15^ins15^*. When co-expressed from a centromeric plasmid, *nhp2^ins9^* and *utp15^ins15^* were actually able to suppress growth defects on glucose in the Ins9 (*ins9*, *rps15-1*, *GAL::RPS15*) and Ins15 (*ins15*, *rps15-1*, *GAL::RPS15*) strains respectively, albeit much less efficiently than the wild-type allele (data not shown). To confirm that these mutations are indeed responsible for the synthetic lethality with *rps15-1*, we crossed the Ins9 and Ins15 strains to either the *Δnhp2::kanMX4*, *rps15-1* or *Δutp15::kanMX4*, *rps15-1* null mutants respectively. The resulting *Δnhp2/nhp2^ins9^*, *rps15-1/rps15-1* diploid strain was not viable in the absence of a complementing wild-type copy of *RPS15*, while the backcross with the *NHP2^+^*, *rps15-1* strain yielded a viable diploid strain. Similar results were obtained with *utp15^ins15^*. We thus conclude that *nhp2^ins9^* and *utp15^ins15^* are indeed mutations synthetically lethal with *rps15-1*.

Complementation cloning in the other *INS* genes was unsuccessful. However, since Ins7 and Ins9 showed very similar molecular phenotypes (see below), we sequenced the *NHP2* locus in Ins7, and found the exact same *nhp2^ins9^* mutation. For simplification, these strain will be referred to as Ins7/9, bearing the *nhp2^ins9^* mutation.

### Phenotypic analysis

**Figure 3:**
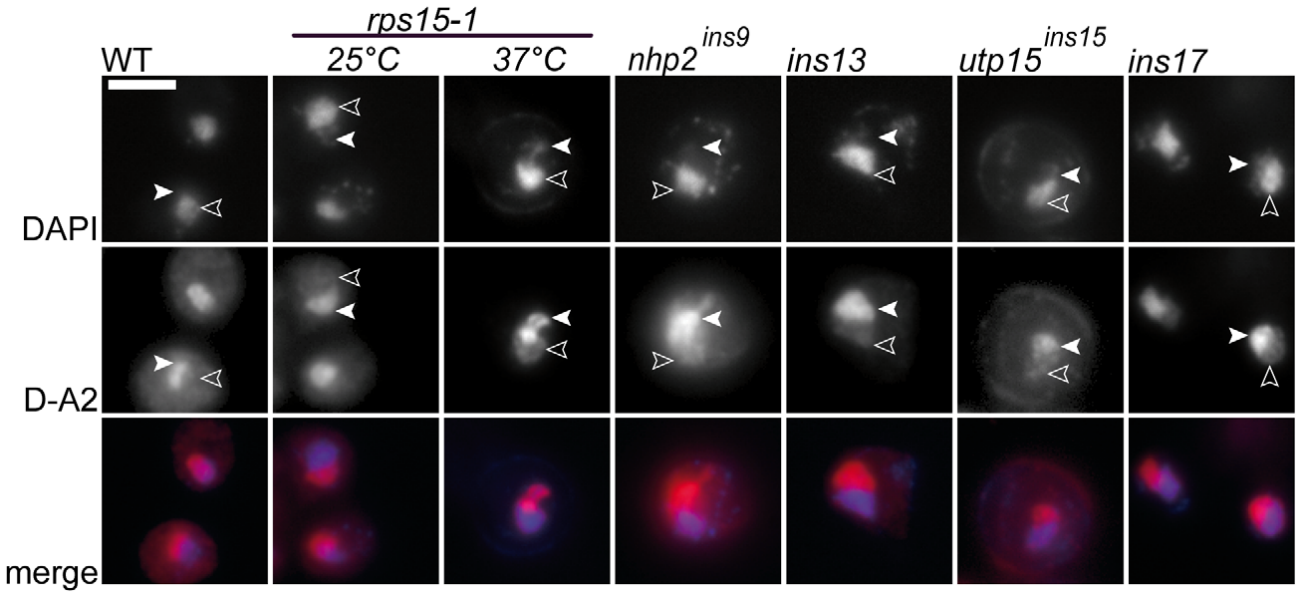
Detection of small subunit ribosomal precursors by fluorescence in-situ hybridization. WT, parental *rps15-1* strain at the indicated temperature, or representatives Ins strains after two generations in glucose containing media were stained with DAPI (upper row) or ITS1-cy3 probe (middle row). Empty triangles point towards DAPI (nucleoplasmic) staining; white triangles point towards ITS1 nucleolar staining. Bar represents 5 µm.

Ribosome biogenesis is arguably a well-ordered and compartmentalized process, as depicted in Figure 1. Early 35S maturation takes place in the nucleolus, where A2 cleavage separates large and small pre-subunit maturation pathways. Pre-40S particles are rapidly exported to the cytoplasm where an endonucleolytic cleavage at site D releases the ITS1 from the 18S rRNA, thus completing maturation. Rps15p was shown to be involved in nuclear export, a late step in ribosome biogenesis [6]. Since mutations synthetic lethal with *rps15-1* were identified in *NHP2* and *UTP15*, two genes coding for proteins known to have a role in the early steps of pre-RNA maturation, we sought to characterize the rRNA sub-cellular distribution in the double mutant strains to understand the origin of the synthetic lethal relationship. By using a D-A2 (ITS1) probe in fluorescence *in situ* hybridization (FISH) experiments, it is possible to visualize all 18S rRNA precursors, as depicted in Figure 1. In wild-type yeast cells, for instance, there is a mild ITS1 signal in both cytoplasm and nucleoplasm, along a strong nucleolar signal (Fig. 3). At 25°C in the presence of glucose, the *rps15-1* reference strain presents essentially a wild-type phenotype (Fig. 3), with lower cytoplasmic accumulation of the D-A2 signal in a small minority of cells. At 37°C, this strain shows a strong nucleolar and nucleoplasmic accumulation (Fig. 3), similar to Rps15p depletion [6]. We analyzed the distribution for the 18S rRNA precursors in mutant cells grown in permissive (Gal) and restrictive (Glc) conditions. Representative pictures for some mutants in restrictive conditions are shown in Fig. 3, and our observations are summarized in Table 1. There is a strong nucleolar signal in restrictive conditions for Ins7/9 and Ins15 consistent with defects in early processing steps. However, persistence of a cytoplasmic signal similar to the one in the *rps15-1* reference strain indicates that pre-40S particles export is still taking place. In contrast, all other Ins strains are devoid of a cytoplasmic ITS1 signal, and display retention of pre-40S particles in the nucleoplasm, which indicates that pre-40S particles are not exported to the cytoplasm.

**Table 1:** Phenotype summaries for Ins strains.

		D-A2 FISH (a)					Northern Blot (b)		
	nucleolus	nucleoplasm	cytoplasm	35S	32S	27SA2	23S	21S	20S
Ins3	+	++	-	+ (c)	=	=	+ (c)	=	=
Ins5	+	++	-	=	=	=	=	=	=
Ins7	++	+	+	+	-	=	+ (c)	=	=
Ins9	++	+	+	+	-	=	+ (c)	=	=
Ins11	+	++	-	=	=	-	+ (c)	+	=
Ins13	+	++	-	=	=	=	=	=	+
Ins15	++	+/−	+/−	-	-	-	-	=	-
Ins17	+	++ (d)	-	=	=	=	=	=	++

(a) **+** same as *rps15^ts^*, **++** accumulation, **-** absence, **+/−** intermediate. Restrictive conditions except where noted ^(d)^.

(b) **= ** comparable to *rps15^ts^*, **+** accumulation, **-** reduction. Permissive and restrictive conditions, except where noted ^(c)^.

(c) only in restrictive conditions.

(d) in restrictive and permissive conditions.

**Figure 4:**
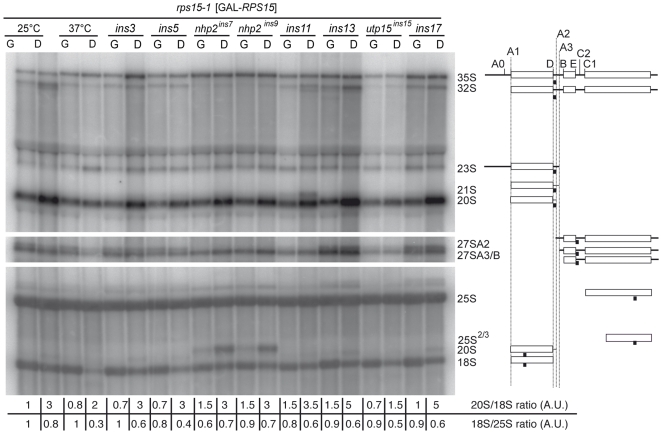
Northern blot of whole rRNAs. Schematic representation and names of the identified RNAs are listed on the right. Pre-rRNAs were detected with probes D-A2 (upper panel), E-C2 (middle panel) and 18S and 25S (lower panel). These probes are depicted as black squares along the identified rRNA species (see table 3 for sequence). Cells were grown as in Fig. 3 in medium containing galactose (G) or glucose (D).

We then characterized defects at the molecular level by Northern Blot, probing whole cellular RNAs with various oligonucleotides depicted in Fig. 4. rRNA signals were normalized to 25S rRNA or 18S rRNA (Fig. 1). These observations are summarized in Table 1. In both permissive and restrictive conditions, the Ins7/9 strains show a strong decrease of the 32S pre-rRNA, while 35S pre-rRNA accumulates. This is paralleled by 23S RNA accumulation and 27SA2 RNA decrease. This phenotype indicates that early cleavages A0, A1, A2 are defective. Regarding Ins15, all rRNAs except 25S rRNA drastically under-accumulate, irrespective of their size, including 35S pre-rRNA, suggesting a defect in rDNA transcription. These pre-rRNA processing defects are consistent with the nucleolar accumulation observed with the D-A2 probe by FISH, and resembles the molecular phenotypes observed upon depletion of Nhp2p or Utp15p: Utp15p was proposed to be required for optimal rRNA synthesis [2], [11], whereas Nhp2p depletion led to an A0-A1-A2 cleavage defect [10]. In glucose, the depleted GAL-*nhp2* strain presented a decreased 20S rRNA level [10] while the Ins7/9 strains described here displayed a steady 20S rRNA level in permissive conditions and an actual increase in restrictive conditions. This suggests a maturation defect additive to that induced by Nhp2 loss of function, likely due to the *rps15-1* mutation, as 20S pre-rRNA accumulation is also observed for the *rps15-1* reference strain (Fig. 4, lane 1 and 2). Similar results are observed with utp15*^ins15^*.

None of the other mutant stains showed remarkable processing phenotypes, except Ins11 which displayed abnormal 21S RNA in parallel with a disappearance of 27SA2 RNA, indicative of defective cleavage at site A2. The Ins17 strain, and to a lesser extent Ins13, showed a much stronger level of 20S rRNA when normalized to 18S (Fig. 4) or 25S rRNAs. However, in contrast to Ins7/9 and 15, pre-40S particles in Ins3, 5, 11, 13 and 17 were strongly retained in the nucleoplasm, which suggests that the mutations involved affect the export competence of these particles, either in *cis* or in *trans*.

Relative to the parental strain, all Ins strains under-accumulated 18S rRNA, while the levels of 25S rRNA did not seem to be affected, proving our screen based on an *rps* mutant to be small subunit specific. We observed however a very unusual form of rRNA with the 25S probe in mutants Ins7/9, building up to high levels in restrictive conditions. We probed this RNA with oligos 25S-01 (complementary to 2347-2377 in 25S rRNA), 25S-02 (1431–1452) and 25S-03 (638–658), of which only 25S-01 and 02 were positive. With an estimated size of around 2,1 Kb, this new rRNA species spans the 3′ two-thirds of the mature 25S rRNA and we therefore named it 25S^2/3^. It is known that absence of some pseudouridylation guide H-ACA snoRNAs can actually perturb rRNA cleavages [12], [13], but we do not know at this point if 25S^2/3^ rRNA is an aberrant processing by-product or a partial 25S rRNA degradation product.

### *bud23* and *slx9* are synthetic lethal with *rps15-1*

As we were not able to identify the other *ins* mutations by complementation experiments due to high reversion rates, we looked at phenotypic analogy with mutations described in the litterature. For instance, Ins17, which shows the strongest 20S rRNA accumulation in the nucleoplasm, is linked to the *MAT* locus as evidenced by analyzing tetrads obtained from the initial backcross to *rps15-1* (data not shown). *BUD23* has been described as being a non-essential methyltransferase responsible for modifying 18S rRNA residue G1575. *BUD23* is closely linked to the *MAT* locus, and deletion for *bud23* leads to a small subunit export phenotype [14] very similar to the one observed for the Ins17 strain even in permissive conditions. This strongly pointed towards *ins17* to be a *BUD23* allele. We first decided to examine whether *bud23* deletion is synthetic lethal with *rps15-1*. A *Δbud23::kanMX4* strain was crossed with an *rps15-1* strain, and the resulting diploid was set to sporulate. G418^r^ thermosensitive spores were incapable of forming colonies, indicating that a *Δbud23*, *rps15-1* genetic combination is synthetically lethal. Sequencing of *BUD23* in the Ins17 strain showed no mutation in the ORF or 250 nucleotides surrounding. Thus *BUD23* is an additional genetic interactant with *RPS15*.

Similarly, Ins11 stood out during our phenotypic analysis, as it showed accumulation of both 23S and 21S rRNA. Based on this phenotype, which was not often described in the literature, we sequenced some candidates in the Ins11 strain, like *RPS19A/B*, *RPS18A/B*, *RPS3*, *YAR1*, *ENP1*, *LTV1*, and *SLX9*, but found that none of them was mutated (data not shown). Because deletion of the non-essential gene *SLX*9 shows a striking phenotypic resemblance with Ins11, with the same rRNA precursors accumulating [15], we wondered if it would also present genetic interactions with *RPS15*. We crossed a *Δslx9::kanMX4* mutant with our *rps15-1* mutant. After sporulation, Δ*slx9*, *rps15-1* spores were incapable to grow, indicating synthetic lethality. Thus, we identified two genes which deletion is synthetic lethal with *rps15-1*, in addition to the mutations identified in our synthetic lethal screen. When attempting to construct a *Δbud23*, *rps15-1* [pFL38-*GAL::RPS15*] strain, recombinant descendants were unviable in Galactose, suggesting that a *Δbud23* deletion is also lethal when Rps15p is over-expressed with a GAL promoter. This could explain why no *BUD23* mutant was isolated in our screen. Moreover, our screen was clearly not saturated, as no additional lethal mutation was selected for in *rps15-1*.

### Are specific rRNA modifications responsible for synthetic lethality?

**Figure 5:**
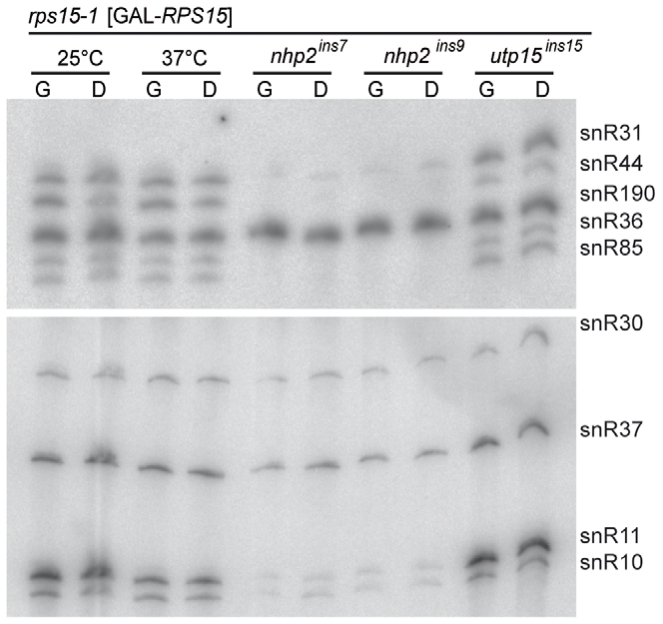
Northern blot of snoRNAs. snoRNA species listed on the right were probed with relevant oligonucleotides as listed in Table 3. Strains and growth conditions (G for galactose, permissive and D for glucose, restrictive) are listed at the top. Cells were grown as in Fig. 3.

*NHP2* and *BUD23*, two of the mutated genes showing synthetic lethality with *rps15-1*, are involved in rRNA modifications, suggesting a functional link between Rps15p and rRNA modifications. Nhp2p is required for synthesis and stability of H/ACA snoRNPs, which are responsible for targeting pseudo-uridylation [10]. We examined the influence of the *nhp2^ins9^* mutation on the state of the H/ACA snoRNAs accumulation (Fig. 5). Abundance of snoRNAs in *nhp2^ins9^* strains was clearly affected, with very low levels of all the H-ACA snoRNA tested, except snR30 and snR37, even in a *RPS15^+^* background (in galactose, G, in Fig. 5), while snR190, a C/D box snoRNA involved in rRNA methylation through a different type of snRNP, remained unaffected.

**Figure 6:**
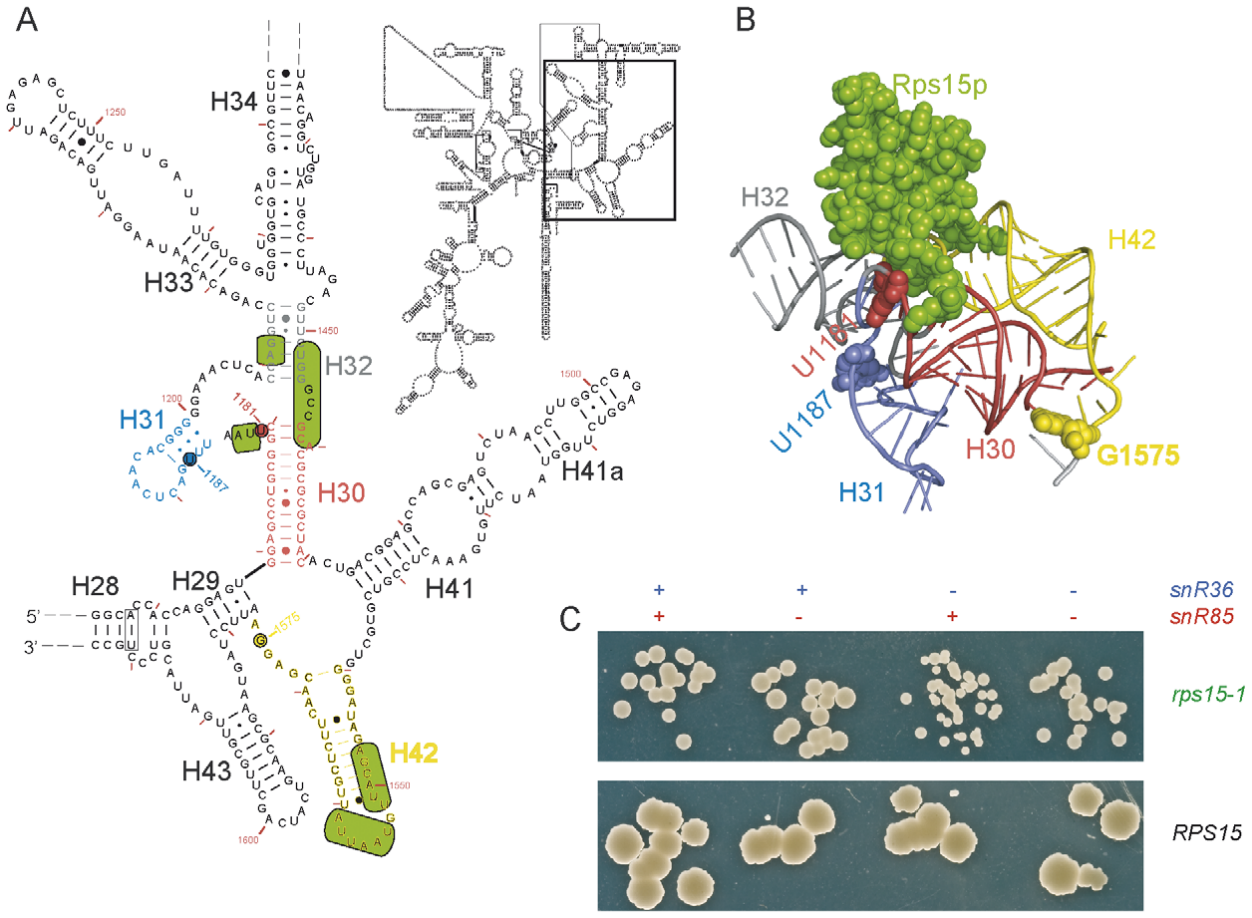
Positions of Rps15p and rRNA modifications on 18S rRNA, and growth phenotypes for snoRNA-deleted mutants. **A**. 2D representation for yeast 18S rDNA sequence. Red : Helix 30, Blue : H31 and loop, Grey : H32 and distal unpaired nucleotides, Yellow : H42 and surrounding unpaired nucleotides. Rps15p contacts (green) with 18S rRNA are extrapolated from those between S19 and 16S rRNA [16]. 2D representation obtained from CRW http://www.rna.ccbb.utexas.edu/ [30] for Genebank sequence #U53879. **B**. 3D representation of the ribosomal region surrounding prokaryotic S19, in green (Rps15p homolog). Colors as above. Equivalences for eukaryotic 18S rRNA U1181 (red, target of snr85), U1187 (blue, target of snr36) and G1575 (yellow, target of Bud23p) are indicated in colored spheres. Obtained using Pymol and PDB #1FJG. **C**. Yeast spotted as single cells. Relevant genotype are indicated at the top concerning snoRNAs and right for *RPS15* background.

Interestingly, a few pseudo-uridines lie near Rps15p's binding site (extrapolated from the bacterial 30S subunit structure [16]), the closest being at positions U1181 (red in Fig. 6A, 6B) and U1187 (blue in Fig. 6A, 6B), guided by snR85 and snR36 respectively. Bud23p is a methyltransferase responsible for 18S rRNA modification, the m^7^G1575 methylation [14]. It is noteworthy that G1575 is located near helix 29 (H29, shown in yellow in Fig. 6A, 6B), while Rps15p's binding to rRNA, as deduced from the position of its bacterial homolog S19 in the prokaryotic 30S crystal structure [16] (green in Fig. 6A, 6B), is located at the H30-H31-H32 junction, only a few Å away.

Remarkably, snR85 and snR36 were almost undetectable in the Ins7/9 strains (Fig. 5). While we do not know if under-accumulation of these snoRNA greatly reduces or abolishes 18S rRNA modifications, we directly questioned whether synthetic lethality between *nhp2^ins9^* and *rps15-1* was due to specific absence of these snoRNAs. We constructed double mutants *rps15-1*, *Δsnr36* and *rps15-1*, *Δsnr85*, as well as the triple *rps15-1*, *Δsnr36*, *Δsnr85* mutant. All genetic combinations were viable, indicating that the synthetic lethal relationship between *rps15-1* and *nhp2^ins9^* cannot be explained by the absence of the snR36 and snR85 guided modifications in the vicinity of Rps15p's binding site. However, although deletion of these snoRNAs displayed no measurable growth defect in a wild-type background (Fig. 6C, bottom row), we observed faster growth when *snr85* was deleted in the *rps15-1* background, while the *snr36* deletion had a synthetic sickness effect with *rps15-1* (Fig. 6C, top row). In liquid culture, generation times were consistent, ranging from 3.8 h for *Δsnr85*, *rps15-1* to 4.5 h for *Δsnr36*, *rps15-1*, with the parental *rps15-1* strain doubling every 4.1 h (data not shown). When *snr36* and *snr85* deletions were combined, both synthetic suppression and synthetic sickness appear to neutralize each other, as this triple mutant grew like the *rps15-1* parental strain (Fig. 6C, top row).

Similar to deletion of snR36 and snR85, a *BUD23* allele encoding an enzymatically inactive protein (G57E or D77K from [14]) was not synthetic lethal with *rps15-1* (data not shown), indicating that the absence of methylation m^7^G1575 is not sufficient to explain synthetic lethality between *Bud23Δ* and *rps15-1*.

Thus, as anticipated from the structure of the 40S subunit, these data suggest a complex interplay between Rps15p and the pseudo-uridylation at position U1181 or U1187. However, synthetic lethality between *nhp2^ins9^* and *rps15-1* appear to be due to a wider impairment of the H/ACA snoRNP function.

## Discussion

We designed a novel approach to perform a synthetic lethal screen aimed at uncovering more precisely the function of Rps15p in ribosome biogenesis. We identified 8 mutants in a specific screening process based on conditional (galactose induced/glucose repressed) expression of the WT allele. Upon functional complementation on glucose (Fig. 2), two mutations were identified in *NHP2* and *UTP15*. The *rps15-1* allele was also found to be synthetic lethal with deletions of *BUD23 or SLX9*. The mutants were assigned to two main classes: those retaining pre-40S particles in the nucleoplasm, which could be genuine mutants in the export pathway, like Ins3, Ins5, Ins13, Ins17, and *Δbud23*; and mutants affected in early, nucleolar steps in ribosome biogenesis, such as Ins11, *nhp2^ins9^*, *utp15^ins15^* and *Δslx9*. In double mutants *rps15-1*, *nhp2^ins9^* and *rps15-1*, *utp15^ins15^*, 20S pre-rRNA is readily detected in the cytoplasm, indicating that pre-40S particles are exported from the nucleus despite upstream defects, even in restrictive conditions. It is possible that overall rRNA processing is slowed down, but can still be pursued up to the final cytoplasmic D cleavage; lethality could arise from a trivial “flow” defect in ribosome production, due to two independent bottlenecks. But more specific hypotheses for the observed synthetic lethality can be envisioned. It has been reported that Utp15p depletion triggers cell cycle arrest [17]. The Ins15 strain stops growth almost immediately in restrictive conditions, merely undergoing one additional division. In this case, it is conceivable that the two hypomorphic mutations *rps15-1* and *utp15^ins15^* provoke a cell cycle arrest in restrictive synthetic conditions, when their defects are additive. Regarding *NHP2* synthetic lethality, numerous snoRNAs deletions have been described as yielding defects in ribosome biogenesis defects causing translational defects [12], and the *rps15-1* mutation affects translational accuracy (GS & FG, unpublished). Additive mutations affecting either ribosome primary function, or subunit imbalance in the cytoplasm, could lead to a strong translational phenotype causing cell death.

Although examples of synthetic lethality between early players and late players in ribosomal small-subunit biogenesis have been identified before, as for *GAR1* and *RIO1* [18], isolation of *NHP2* and *UTP15* as *RPS15* genetic interactors was quite unexpected. Moreover, a good part of the synthetic lethal mutants isolated here accumulate early 21S or 23S rRNA precursors, while Rps15p depletion was shown to result in 20S pre-RNA accumulation. Rps15p could be involved in several steps during ribosome biogenesis. Rps15p depletion [6] or the *rps15-1* mutant at permissive temperature affect the export function, but early nucleolar steps could be affected in the *rps15-1* mutant especially at restrictive temperature, as suggested by the accumulation for 23S rRNA precursor in the *rps15-1* strain at restrictive temperature (Fig. 4, lane 4). Such phenotypic discrepancies have already been revealed by studies on *RPS14*, showing a strikingly different phenotype for depletion of the whole protein causing an early small subunit processing blockage [19], or point mutations leading to late high-level 20S cytoplasmic accumulation [20]. However, *RPS15* depletion induces a late maturation defect, while the *rps15-1* recessive mutant would display an early processing phenotype, which seems counter-intuitive. So reciprocally, it is possible that although hypomorphic mutations in Utp15p and Nhp2p, whose depletion results in early ribsome biogenesis defects, rather perturb downstream steps in the pathway. For instance, specific snoRNAs shortage in the *nhp2^ins9^* mutant might have consequences more downstream in conjunction with *rps15-1*. In this respect, we tried to identify specific synthetic lethality between *rps15-1* and a subset of H-ACA snoRNA guides targeted close to the binding site of Rps15p on 18S rRNA, driven by snrR36 and snR85. This idea was strengthened by the finding of a synthetic lethal interaction between *rps15-1* and absence of Bud23p, which methylates G1575 in 18S rRNA, close to Rps15p's binding site. Although deletion of the two snoRNAs resulted either in synthetic sickness with *rps15-1* or partial suppression of *rps15-1* growth impairment, it could not explain the *nhp2^ins9^* synthetic lethality with *rps15-1*.

Nevertheless, the synthetic phenotype presented by either snoRNA deletion is striking, for such a synthetic sickness (*Δsnr36*) or partial suppression (*Δsnr85*) has never been reported for a single snoRNA to our knowledge. Although additive effects were observed for combinations of snoRNA deletions [12], [21], opposite phenotypes that suppress each other, as observed for *Δsnr36* and *Δsnr85* in the *rps15-1* background, have never been shown before. This effect might be explained in part by the fact that the targets of these snoRNAs overlap on the 18S rRNA, making it impossible for both snoRNAs to intervene simultaneously. Also, in a bacterial in vitro reconstituted system, the binding of S19 to 16S rRNA was reported to sustain a conformational switch, which involves H30 and H31 [22]. These two helices are targeted in yeast by snR85 and snR36, which could affect in opposed way this conformational rearrangement, triggered by S19 homolog, Rps15p. Our data thus give hints on the interplay between a ribosomal protein and snoRNA mediated modifications that significantly impair ribosome biogenesis and/or function. Such an approach could be extended to identify other specific interactions between ribosomal protein mutants and snoRNA deletions to help understand the role of rRNA modifications, which are still largely unknown.

Finally our most intriguing result is the accumulation of an aberrant large subunit 25S^2/3^ rRNA form in the *nhp2^ins9^* mutant, when Rps15p is mutated and thus limits small subunit availability. It raises the possibility that a 60S/40S imbalance leads to degradation of the large subunit. In an *nhp2^ins9^* mutant, such a degradation process could be stalled on the hypomodified 25S rRNA. This degradation pathway could involve the TRAMP complex and the exosome [23] or the recently described ubiquitine-mediated NRD (Non-functional Ribosome Decay) [24]. How absence of rRNA modifications could perturb degradation remains to be understood. Accumulation at high levels of this stalled degradation product could actually be an explanation for synthetic lethality between *rps15-1* and *nhp2^ins9^*, and be a genetic starting point to identify actors in this pathway.

## Materials and Methods

### Yeast strains and media

Regular yeast genetic methods were used [25]. Relevant *Saccharomyces cerevisiae* strains are listed in Table 2 and can be obtained on demand to the corresponding author. Cells were grown either in YP medium (1% yeast extract, 1% peptone) supplemented with 2% galactose or 2% glucose as the carbon source, or in YNB medium (0.17% yeast nitrogen base, 0.5% (NH_4_2SO_4_) supplemented with 2% galactose or 2% glucose and the required amino acids and bases. When required, G418 and doxycyclin were added at 0.2 mg/ml and 30 µg/ml final concentrations, respectively.

**Table 2:** Yeast strains used in this work.

Strain	Relevant Genotype	Source
BY4741	*MATa his3Δ1 leu2Δ0 met15Δ0 ura3Δ0*	Euroscarf WT
ME14-i3	*MATa his3Δ1 leu2Δ0 lys2Δ0 ura3Δ0 rps15-1*	This work a)
Ins x	*MATa his3Δ1 leu2Δ0 lys2Δ0 ura3Δ0 rps15-1 insx [pFL38-GAL::RPS15]*	ME14-i3 UV-mutagenized
ME17-F4	*MATα Δhis3Δ1 leu2Δ0 ura3Δ0 lys2Δ0 Δnhp2::kanMX4 [pCM189-NHP2]*	Euroscarf Y23906 spore
ME17-F7	*MATα Δhis3Δ1 leu2Δ0; ura3Δ0 lys2Δ0 Δutp15::kanMX4 [pCM189-UTP15]*	Euroscarf Y26228 spore
YO7184	*MATa his3Δ1 leu2Δ0 met15Δ0 ura3Δ0 Δbud23::KanMX4*	Euroscarf
YO4711	*MATa his3Δ1 leu2Δ0 met15Δ0 ura3Δ0 Δslx9::KanMX4*	Euroscarf
LMA458	*MATα his3Δ1 leu2Δ0 lys2Δ0 ura3Δ0 snr85::KAN*	Kindly provided by A. Jacquier and M. Fromont-Racine [28]
ME17-i5	*MATα his3Δ1 leu2Δ0 lys2Δ0 ura3Δ0 snr85::KAN rps15-1*	ME14-i3 x LMA458 spore
ME17-i6	*MATa his3Δ1 leu2Δ0 met15Δ0 ura3Δ0 snr36::URA3*	BY4741 b)
ME17-i8	*MATa his3Δ1 leu2Δ0 lys2Δ0 ura3Δ0 snr36::URA3 rps15-1*	ME14-i3 b)
ME18-a1	*MATα his3Δ1 leu2Δ0 lys2Δ0 ura3Δ0 snr85::KAN snr36::URA3*	LMA458 b)
ME18-d6	*MATα his3Δ1 leu2Δ0 lys2Δ0 ura3Δ0 snr85::KAN snr36::URA3 rps15-1*	ME17-i5 x ME17-i8 spore

a) strain GAL-RPS15 [6] transformed with a PCR holding the *rps15-1* allele (kindly from P. Milkereit, unpublished). A thermo-sensitive clone was isolated and cured from pFL38-GAL::RPS15 plasmid. *rps15-1* locus was sequenced for confirmation.

b) transformed with PCR product amplified from the Δ*snr36::URA3* strain from [29], kindly provided by E. Fayet-Lebaron. Absence of SnR36 was checked by Northern blotting.

### Plasmids

pFL38-*RPS15wt* or pFL38-*rps15-1* contain the promoter and coding sequence for *RPS15* or *rps15-1*, followed by a PGK terminator (Valérie Choesmel-Cadamuro, unpublished). pFL38-Gal::RPS15wt was described earlier [6]. pCM189-NHP2 and pCM189-UTP15 are based on ARS-CEN, URA3 pCM189 [26], with the cloned ORF under control of the TET promoter, repressed in presence of doxycyclin. pRS315 [27] and pAJ2154, 2155 and 2156 were described previously [14] and kindly provided by R. Sardana.

### Oligos and probes

Oligonucleotides sequences used to construct strains and plasmids can be obtained on demand to the corresponding author. Oligonucleotides used for FISH or northern blot are listed in Table 3.

**Table 3:** Oligonucleotide probes used in this work.

Name	Sequence (5′ 3′)
D-A2	TTG CAC AGA AAT CTC TCA CCG TTT GGA ATA GCA AGA AAG AAA CTT ACA AGC TT
18S-01	TTG TTC CTC GTT AAG GTA TTT ACA TTG TAC TCA TTC C
25S-01	CCC GTT CCC TTG GCT GTG GTT TCG CTA GAT A
25S-02	TGC TAC TAC CAC CAA GAT CTG C
25S-03	CTT GGT CCG TGT TTC AAG ACG
E-C2	ATA GGC CAG GAA TTT CAA GTT AAC TCC AAA GAG TAT CAC TC
snR10	CAT GGG TCA AGA ACG CCC CGG AGG GG
snR11	GAC GAA TCG TGA CTC TG
snR30	GAA GCG CCA TCT AGA TG
snR31	GTAGAACGAATCATGACC
snR36	TTA CTC GAG TGA TAT GAG ACG TTC TAA TTA
snR37	GAT AGT ATT AAC CAC TAC TG
snR44	GCC CGA TCA CAC CAT CTA GTT ATC AGC CCG
snR85	GAC ATA TGT GCT AGT ATT CG
snR190	CGA GGA AAG AAG AGA CAC CAT TAT C

### Fluoresence *In Situ* hybridization

Detection of pre-rRNAs by FISH was performed as described [4], using ITS1 (D-A2) oligonucleotidic DNA probe TT*GCACAGAAATCTCT*CACCGTTTGGAAT*AGCAAGAAAGAAACT*TACAAGCT*T, where T* is an amino-modified deoxy-thymidine conjugated to Cy3. DNA was stained with DAPI. Images were captured with a CoolSnap CCD camera (Photometrics) mounted on a DMRB microscope (Leica) and processed with Metamorph software (Universal Imaging).

### Northern Blotting

Total RNAs were prepared from yeast cells using Trizol (Invitrogen), essentially as described by the manufacturer. Briefly, 25 ml of cell culture at 1-2×10^7^ cells/ml were centrifuged and the pelleted cells were washed once in water, resuspended in 0.5 ml Trizol (Invitrogen) with 200 µl acid-washed glass beads (0.4–0.5 mm diameter) in a 2 ml eppendorf tube, and mixed with a vortex for 6 min at 4°C. After 5 min at 65°C and 5 min at room temperature, 200 µl Chloroform were added, followed by mixing for 15 sec. After 3 min at room temperature and 15 min centrifugation at 13′000 g and at 4°C, 400 µl of aquous phase were precipitated for 10 min at room temperature in a fresh tube with 500 µl isopropanol. RNA was pelleted 15 min at 13′000 g, 4°C, washed once with cold 70% ethanol, centrifuged for 5 min at 13000 g, 4°C, and then dried at room temperature after supernatant removal. RNAs were resuspended in 50 µl formamide and heated for 5 min at 55°C.

Three µg RNA was fractionated on a 1% agarose gel in 30 mM triethanolamine, 30 mM tricine, and 1.25% formaldehyde, for large RNA species, or 6% polyacrylamide/urea gels in TBE (0.89 M Tris base, 0.89 mM boric acid, and 20 mM EDTA) for snoRNAs, and then transferred to a Hybond-N+ membrane (GE Healthcare) in 0.5x TBE buffer by passive transfer or semi-dry electrotransfer (Trans-Blot SD, BioRad), followed by UV cross-linking to the membrane. Blots were hybridized with 5′ ^32^P-labeled oligodeoxynucleotides (Table 3) using RapidHyb Buffer (Amersham).
